# Prognostic Value of Cheyne-Stokes Respiration and Nutritional Status in Acute Decompensated Heart Failure

**DOI:** 10.3390/nu15040964

**Published:** 2023-02-15

**Authors:** Abidan Abulimiti, Ryo Naito, Takatoshi Kasai, Sayaki Ishiwata, Miho Nishitani-Yokoyama, Akihiro Sato, Shoko Suda, Hiroki Matsumoto, Jun Shitara, Shoichiro Yatsu, Azusa Murata, Megumi Shimizu, Takao Kato, Masaru Hiki, Hiroyuki Daida, Tohru Minamino

**Affiliations:** 1 Department of Cardiovascular Biology and Medicine, Juntendo University Graduate School of Medicine, Tokyo 113-8421, Japan; 2 Cardiovascular Respiratory Sleep Medicine, Juntendo University Graduate School of Medicine, Tokyo 113-8421, Japan; 3 Sleep and Sleep-Disordered Breathing Center, Juntendo University Hospital, Tokyo 113-8421, Japan; 4 Department of Cardiovascular Management and Remote Monitoring, Juntendo University Graduate School of Medicine, Tokyo 113-8421, Japan; 5 Faculty of Health Science, Juntendo University, Tokyo 113-8421, Japan; 6 Japan Agency for Medical Research and Development-Core Research for Evolutionary Medical Science and Technology (AMED-CREST), Japan Agency for Medical Research and Development, Tokyo 113-8421, Japan

**Keywords:** acute decompensated heart failure, Cheyne-Stokes respiration, nutritional status

## Abstract

Malnutrition frequently coexists with heart failure (HF), leading to series of negative consequences. Cheyne–Stokes respiration (CSR) is predominantly detected in patients with HF. However, the effect of CSR and malnutrition on the long-term prognosis of patients with acute decompensated HF (ADHF) remains unclear. We enrolled 162 patients with ADHF (median age, 62 years; 78.4% men). The presence of CSR was assessed using polysomnography and the controlling nutritional status score was assessed to evaluate the nutritional status. Patients were divided into four groups based on CSR and malnutrition. The primary outcome was all-cause mortality. In total, 44% of patients had CSR and 67% of patients had malnutrition. The all-cause mortality rate was 26 (16%) during the 35.9 months median follow-up period. CSR with malnutrition was associated with lower survival rates (log-rank *p* < 0.001). Age, hemoglobin, albumin, lymphocyte count, total cholesterol, triglyceride, low-density lipoprotein cholesterol, creatinine, estimated glomerular filtration rate, B-type natriuretic peptide, administration of loop diuretics, apnea-hypopnea index and central apnea-hypopnea index were significantly different among all groups (*p* < 0.05). CSR with malnutrition was independently associated with all-cause mortality. In conclusion, CSR with malnutrition is associated with a high risk of all-cause mortality in patients with ADHF.

## 1. Introduction

In developed countries, heart failure (HF) counts as a major public health problem, due to its ability to absorb vast medical resources. As the risk of HF increases with age, HF is predicted to become more prevalent in Japan by 2030 [1,2]. This syndrome along with its complexity has various causes and clinical/hemodynamic profiles but is invariably characterized by elevated left ventricular end-diastolic pressure (LVEDP), increased extracellular fluid volume and symptoms of dyspnea. One more well-recognized sign of HF decompensation is the type of central apnea known as Cheyne–Stokes respiration (CSR). According to the original description by Cheyne [3], CSR is a form of periodic breathing. The ventilatory period is characterized by a prolonged crescendo–decrescendo breathing pattern of tidal volume, followed by apnea or hypopnea. This is assumed to arise as a consequence of the increased chemosensitivity in the setting of growing LVEDP and elevated circulatory time. Most evidence indicates that CSR is related to an increased risk of morbidity and mortality in patients with HF [4].

Recent evidence indicates that malnutrition is linked to poor prognosis in patients with chronic HF [5,6,7]. The pathophysiology of malnutrition involves a catabolic wasting state associated with inflammation and coincident neurohormonal activation, which are frequently observed in HF. Malnutrition may also be a driving factor for HF development, consisting of a domino effect related to autonomic nerve dysfunction and cachexia [7]. However, the setting of acute decompensated HF (ADHF) is complex. ADHF is an acute condition in which symptoms occur newly or alleviate chronic HF. Acute conditions may adjust the baseline nutritional status of patients with ADHF. The Controlling Nutritional Status (CONUT) score is a screening tool which was originally developed to predict acute worsening in surgical patients [8] and thereafter used to identify undernourished patients among the hospitalized population and chronic HF. High CONUT score reportedly has a prognostic impact on patients with chronic cardiac disease [9], including those with ADHF [10,11].

In hospitalized patients with HF following acute decompensation, CSR is observed more frequently than chronic HF in association with higher left ventricular filling pressure and more obvious pulmonary congestion [12]. Patients with ADHF usually have increased sympathetic nerve activity [13] and existing CSR may enhance sympathetic nerve activation [14] and lead to poor prognosis compared to those without CSR. Indeed, patients with ADHF and sleep apnea, including CSR, have a poor prognosis [15]. Malnutrition is also a prognostic factor for patients with HF. Because patients with HF and CSR are generally older, less obese, more congested and frequently use diuretics, all of which may be associated with malnutrition, compared with those without sleep apnea or obstructive sleep apnea [12,16], there might be an interaction between malnutrition and CSR in terms of prognosis. However, the association between CSR and malnutrition in terms of prognosis in patients with ADHF has not been examined. Therefore, we hypothesized that malnutrition in hospitalized patients with HF following acute decompensation with CSR could predict long-term prognosis independent of other known prognostic factors.

## 2. Materials and Methods

### 2.1. Subjects

**Figure 1:**
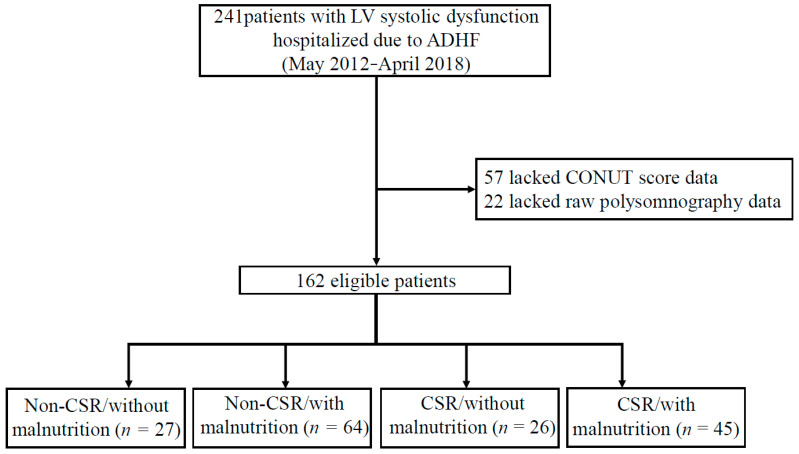
Flow diagram of the study population. From May 2012 to April 2018, 241 patients with LV systolic dysfunction (defined as LVEF < 50% via echocardiography) due to ADHF were hospitalized at Juntendo University Hospital. Among them, 79 patients were excluded for the following reasons: lacked CONUT score data (albumin, total cholesterol) and raw data of polysomnography. Thus, 162 eligible patients were enrolled in the study. Abbreviations: ADHF, acute decompensated heart failure; CSR, Cheyne-stokes respiration; CONUT, controlling nutritional status; LV, left ventricular.

This observational study included data from May 2012 to April 2018 from 241 patients with ADHF who had enrolled in our previous study [15]. Of these, patients who lacked CONUT score data, including missing albumin and total cholesterol data and raw polysomnography data, were excluded. After exclusion criteria, a total of 162 patients were divided into four groups (Non-CSR without malnutrition; Non-CSR with malnutrition; CSR without malnutrition; CSR with malnutrition) according to CSR and malnutrition status (Figure 1). HF following acute decompensation was defined according to the modified Framingham criteria [17]. Diabetes mellitus (DM) was defined as a previous diagnosis from medical records, a hemoglobin A1c value (as calculated by the National Glycolic Hemoglobin Standardization Program) of ≥6.5%, or treatment with oral antidiabetic drugs or insulin. All patients were tracked from the date of admission until April 2019 and were followed up at Juntendo university hospital every one–two month for a median of 35.9 months. Outcome data were obtained by reviewing the electronic medical records of our hospital. The endpoint of interest was the all-cause mortality until April 2019. The Institutional Review Board of Juntendo University Hospital approved the study protocol (Approval no. 871), which abides by the Declaration of Helsinki. Informed consent was obtained from all the patients.

### 2.2. Sleep Study

**Figure 2:**
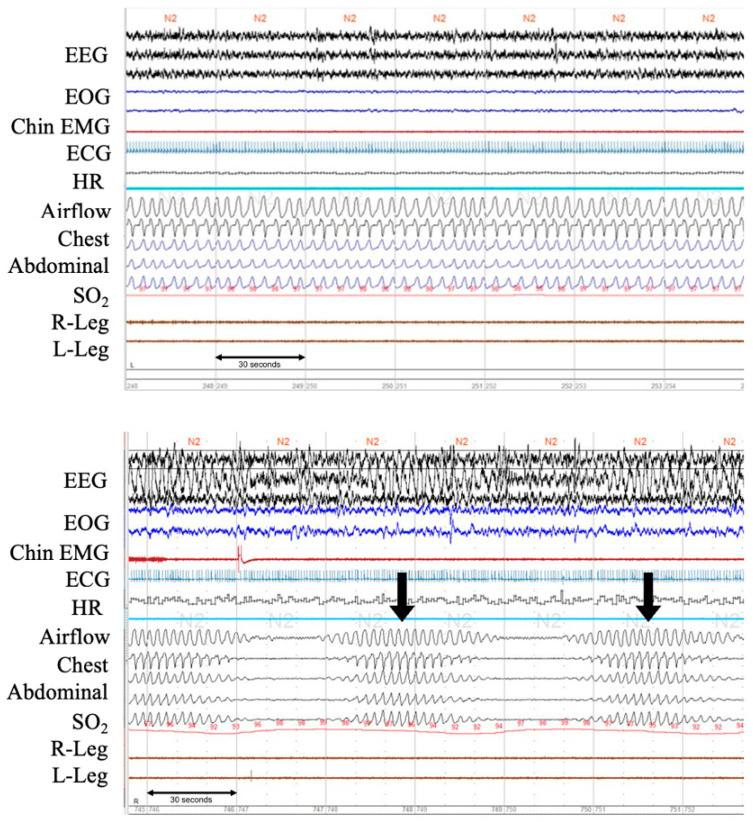
Polysomnographic recordings of normal breathing pattern and a clear pattern of CSR from patients with ADHF. The upper panel shows normal respiration during stage 2 sleep. The lower panel shows Cheyne-Stokes respiration with central sleep apnea. Note in-phase gradual crescendo and decrescendo of tidal volume during hyperpnea and only minimal O_2_ desaturation during hypopnea. EEG, electroencephalogram; EOG, electrooculogram; EMG, electromyogram; ECG, electrocardiogram; HR, heart rate; SO_2,_ oxygen saturation. Arrows (↓) indicate arousals.

All patients underwent overnight complete polysomnography using an Alice PDX (Philips Respironics, Murrysville, PA, USA) for several days after a primary improvement in acute signs and symptoms of decompensation during the initial hospitalization for ADHF. Definitions and scoring methods were according to the American Academy of Sleep Medicine manual version 2.2 [18]. Electrocardiography, electroencephalography, electrooculography and electromyography were performed and thoracoabdominal movement was monitored using respiratory induction plethysmography. Airflow was measured with a nasal pressure cannula and an oronasal thermal airflow sensor. Oxyhemoglobin saturation was monitored using oximetry as described in our previous study [15]. Because the presence or absence of CSR is not formally and specifically reported in our clinical report on polysomnography (only focusing on central sleep apnea), we used the raw polysomnography data to re-score the presence or absence of CSR. CSR was determined as the recording showing at least three consecutive central events with a typical crescendo–decrescendo breathing pattern, with at least five events per hour and a minimum CSR cycle length of ≥40 s or more [19]. Representative raw waveforms of CSR on polysomnography were shown in Figure 2. This was visually scored by two experienced technicians, with the sleep technician’s scoring being overread by a board-certified sleep medicine physician who is certified by the Japanese Society of Sleep Research. Patients were categorized into two groups based on the prevalence of CSR (non-CSR and CSR).

### 2.3. Assessment of Nutritional Status

We assessed nutritional status at admission using the CONUT scoring system to estimate the values of nutrition-related prognostic risk factors. The score was derived from the values of serum albumin, total cholesterol and lymphocyte count. Albumin represents protein reserves, total cholesterol represents caloric depletion and lymphocyte count represents immune defense. A decrease in each component was assigned a high score. Thus, a higher score is closely linked to malnutrition [9,10,20]. CONUT score was calculated according to the original study [19]. We defined patients without malnutrition as CONUT score 0–1 point (normal) and with malnutrition as 2–12 points (including mild, moderate and severe), which is similar to a recent study classification method [21].

### 2.4. Statistical Analysis

Continuous variables are expressed as mean ± standard deviation or median with interquartile range. Categorical variables are presented as numbers and percentages. To compare the baseline characteristics among the groups, one-way ANOVA with Tukey’s post hoc test was used for normally distributed continuous variables and the chi-squared test or Fisher’s exact test was used for categorical variables. The Kruskal–Wallis H test with post-hoc and Mann–Whitney U tests for skewed distributed variables were used for continuous variables. Because of the skewed distributions of age, creatinine and B-type natriuretic peptide (BNP) levels, they were taken into natural logarithm transformations before Cox proportional hazards regression analysis. Cumulative survival curves, drawn using the Kaplan–Meier method, were used to compare the prognosis of the CSR, malnutrition and among the four combined groups using a log-rank test. Univariate and multivariate Cox regression analyses were performed to evaluate the independence of CSR with malnutrition in predicting all-cause mortality during follow-up after accounting for age and creatinine and BNP levels. Hazard ratios (HRs) and 95% confidence intervals (CIs) were calculated. First-order interactions in the multivariate Cox regression analysis were performed by entering interaction terms between the presence or absence of CSR and malnutrition status. The two-sided differences were considered statistically significant at a significance level of 0.05. All analyses were performed using the Statistical Package for Social Sciences software, version 27.00 (Statistical Package for Social Sciences Inc., Chicago, IL, USA).

## 3. Results

### 3.1. Baseline Characteristics

Data from 162 patients were analyzed in this study (Figure 1). Older adults with ADHF (median age, 62 (23) years; 78.4% men) were enrolled. The patient demographics of these four groups are presented in Table 1. There were significant differences in age, hemoglobin, albumin, lymphocyte count, total cholesterol, triglyceride, low-density lipoprotein cholesterol, creatinine, estimated glomerular filtration rate, BNP, administration of loop diuretics, apnea-hypopnea index (AHI) and central apnea-hypopnea index (CAHI) among the four groups (all *p* < 0.05) (Table 1). However, there were 49 (30.2%) ADHF patients who have used positive airway pressure (PAP) therapy and have not shown significant differences across groups. Among them, 22 (13.6%) used continuous PAP therapy and 27 (16.7%) used adaptive servo-ventilation (ASV) therapy.

### 3.2. Separate Prognostic Value of CSR and Malnutrition

**Figure 3:**
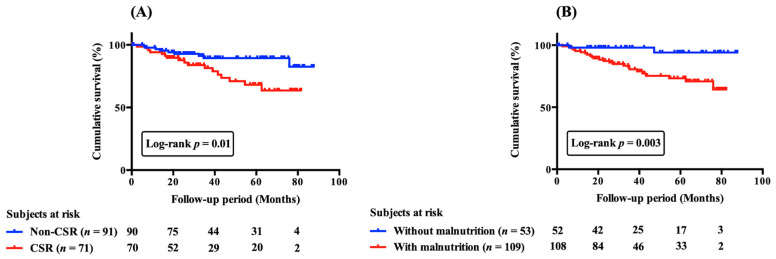
Cumulative survival curves of CSR (**A**) and malnutrition (**B**) on all-cause mortality in patients with acute decompensated heart failure. Cumulative survival curves were significantly different (**A**) between patients with and without CSR and (**B**) between those with and without malnutrition. CSR, Cheyne-stokes respiration.

During a median follow-up of 35.9 (41.5) months, 26 (16.0%) patients died. According to Kaplan–Meier curves (Figure 3A,B), patients with CSR and malnutrition had worse cumulative survival than their counterparts. In univariate analyses, patients with CSR or malnutrition had a greater risk of mortality (HR, 2.58; 95% CI, 1.15–5.79; *p* < 0.05 and HR, 6.31; 95% CI, 1.49–26.75; *p* < 0.05, respectively). Multivariate analysis in model 1, after adjusting for age, creatinine and BNP, demonstrated that either CSR or malnutrition was a significant independent predictor of mortality (HR, 3.92; 95% CI, 1.64–9.39 and 95% HR, 5.77; CI, 1.33–25.00, respectively). However, there was no interaction between the presence or absence of CSR and the presence or absence of malnutrition in terms of prognosis (*p* = 0.35).

### 3.3. Effect of CSR with and without Malnutrition on Prognosis

**Figure 4:**
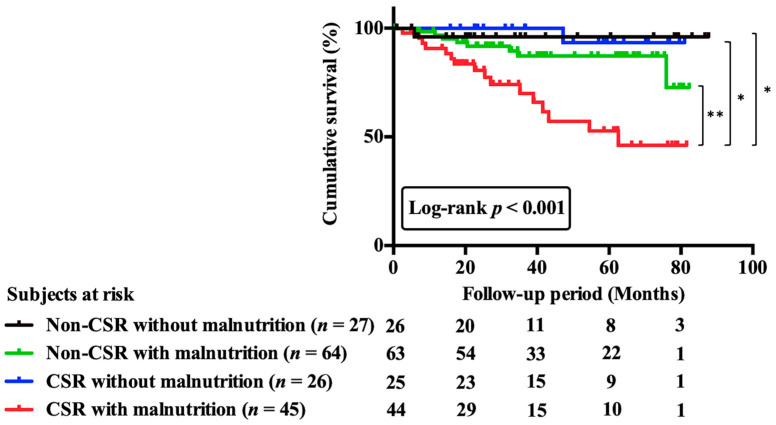
Cumulative survival curves across 4 groups including patients with and without CSR or malnutrition. Patients were categorized into four groups on the basis of prevalence or absence of CSR and of presence or absence of malnutrition. Cumulative survival curves across 4 groups were significantly different. Abbreviations: CSR, Cheyne-stokes respiration. * *p* < 0.05; ** p < 0.01.

Kaplan–Meier curves for cumulative survival and comparison across the four groups are shown in Figure 4. In the univariate analysis, patients in the CSR with malnutrition group had a significantly higher risk of mortality than those in the non-CSR without malnutrition group (HR, 10.76; 95% CI, 1.42–81.35; *p* = 0.02).

Even in multivariate analysis, after accounting for confounding variables in model 2, only CSR with malnutrition (HR, 9.30; 95% CI, 1.23–70.47; *p* = 0.03) was associated with long-term mortality (Table 2).

## 4. Discussion

The present study demonstrated that the presence of CSR in hospitalized patients with HF following acute decompensation was associated with poor long-term prognosis, especially under the presence of malnutrition. Our findings further confirm the importance of sleep or nutritional assessment even in hospitalized patients following acute decompensation and suggest that nutritional assessment in conjunction with early sleep assessment may help identify patients with ADHF who are at high risk of long-term mortality.

### 4.1. CSR and All-Cause Mortality in ADHF Patients

In ADHF, the sympathetic nervous system and the neurohormonal axis, including the renin-angiotensin-aldosterone system, are markedly stimulated [22]. When ADHF combines with CSR, these features are more obvious because of the nature of CSR. The sympathetic stimulatory effects of CSR are not isolated to sleeping but also carry over into wakefulness [13,14]. Daytime plasma norepinephrine concentration and muscle sympathetic nerve burst frequency are significantly higher in patients with ADHF with CSR than in those without CSR [14,23]. Thus, there is a bidirectional correlation between HF and CSR in which the sympathetic nervous system is an important mechanistic intermediary. The presence of CSR in patients with HF is associated with increased morbidity, mortality and impaired quality of life [24,25]. Similarly, poor long-term outcomes were observed in our study. Furthermore, we observed that in hospitalized patients following ADHF, CSR, especially in conjunction with malnutrition, showed an increased risk of all-cause mortality during 35.9 months of the follow-up period.

### 4.2. Malnutrition and All-Cause Mortality in ADHF Patients

A previous study by Narumi et al. suggested that malnutrition is frequently observed in patients with HF. A total of 69% of this patient group has presented with malnutrition, regardless of age, sex, or LVEF [20]. Similarly, in our study, 67% of the study population was diagnosed with malnutrition. Regarding previous outcome research, Kato and Cheng’s investigation in patients hospitalized for ADHF demonstrated that malnutrition is independently associated with in-hospital [26] and long-term mortality [27]. Likewise, in our study, patients with ADHF and malnutrition also had high all-cause mortality during the long-term follow-up. Several studies have shown an association between a single nutritional indicator, including BMI, total cholesterol, serum albumin and total lymphocyte count, and poor outcomes in patients with HF [28,29]. However, the assessment of only one indicator of malnutrition may not provide adequate prognostic information. Several malnutrition indices have been applied in recent studies that can comprehensively evaluate nutritional status in patients with HF, such as the geriatric nutritional risk index (GNRI), prognostic nutritional index (PNI) and CONUT score [26,27].

The CONUT score, a sum of immunity status, protein reserve and caloric depletion, has a significant impact on patients with ADHF [26]. A higher CONUT score implies malnutrition and impaired inflammatory response, supporting the notion that catabolic wasting and the immune system play an essential role in the development of ADHF. Previous Japanese population-based studies have shown that the median BMI was 22 kg/m^2^ in patients with HF [8,28]. In the present study population, the median BMI was >24 kg/m^2^ with and without malnutrition. Furthermore, approximately 35% of malnourished patients with ADHF, regardless of the presence or absence of CSR, had DM, which was higher than that in patients without malnutrition (approximately 15%). Although it is fair to know that malnutrition is commonly prevalent in elderly patients and was associated with all-cause mortality, notably, when interpreting patients under 70 years, the most probable explanation is overnutrition and the prevalence of DM. These results are consistent with previous reports that demonstrated an association between a higher prevalence of DM and malnutrition [29]. The potential effects of DM may be the reason for the relatively higher BMI in our study. This result is similar to that reported by Mineoka et al. [30], which cannot explain malnutrition in patients with higher BMI by the conventional interpretation of weight loss due to malnutrition.

### 4.3. Clinical Implications

Taking everything into consideration, it is crucial to assess nutritional status in conjunction with the presence or absence of CSR inpatients with ADHF from an early stage after admission. Malnutrition may progress to cardiac cachexia, a global wasting process that affects all body compartments [31]. The causes of cachexia in HF are multifactorial and may arise from malnutrition, impaired protein and calorie balance, pro-inflammatory immune activation, neurohormonal derangement, physical deconditioning and prolonged immobilization, leading to catabolic/anabolic imbalance [32,33]. On the other hand, the high prevalence of CSR in patients with ADHF had a significant impact on all-cause mortality, which can challenge cardiologists for disease management. A sleep study in hospitalized patients with HF following acute decompensation is not easy to execute and is not performed regularly. Therefore, the assessment of CSR by PSG, in addition to regular nutritional assessment with ADHF, enables early identification and precise characterization of at-risk patients. Future studies should focus on whether better use of available treatments might improve both CSR and nutritional status and, eventually, outcomes in at-risk patients with ADHF.

### 4.4. Study Limitations

This study has a few limitations. First, the study was a single center study and the study population was relatively small. To assess the association between CONUT score and CSR on the prognosis of patients with HF following acute decompensation, a large sample size and multicenter studies are needed. Second, we did not use the GNRI and PNI for the evaluation of malnutrition status since the study showed that the CONUT score has a better predictive effect on the outcome than the other two [17]. Third, the CONUT score may be reflected by the low plasma cholesterol level resulting from statin therapy. However, the benefits of statins for HF are dubious. Fourth, body fluid volume may affect serum albumin levels in patients with ADHF since blood samples were examined during the acute phase. Fifth, because we excluded patients with HF with a preserved ejection fraction, the results of our study are not applicable to these patients. Sixth, the prognostic effect of PAP therapy under the presence of CSR and malnutrition has not been investigated in our study because no studies suggested the positive prognostic impact of PAP (CPAP or ASV) therapy in patients with HF and sleep apnea [34,35]. Thus, the effects of PAP therapy on sleep apnea in the present study are regarded as a minimum. Therefore, further studies are needed to identify the benefit of PAP in patients with CSR and malnutrition. Last, since the clinical events’ numbers are small, which was related to the small number of subjects, this led to limited statistical power for detecting differences in outcomes among the four groups. In particular, the low event rate in the analysis of the relationship between compliance status and clinical outcome should be interpreted with caution.

## 5. Conclusions

Nutritional status at admission and the presence of CSR in patients with HF following acute decompensation are related to long-term all-cause mortality. In addition to the assessment of nutritional status at admission, the detection of CSR by sleep study may help to identify patients who are at high risk of all-cause mortality in ADHF and result in better care. Nevertheless, further research is warranted to elucidate whether treatment with CSR and malnutrition can prevent mortality and help identify patients most likely to benefit from treatment.

## Figures and Tables

**Table 1:** Characteristics of the study subjects.

Characteristics	All (*n* = 162)	Non-CSR without Malnutrition	Non-CSR with Malnutrition	CSR without Malnutrition	CSR with Malnutrition	*p*-Value *
		(*n* = 27)	(*n* = 64)	(*n* = 26)	(*n* = 45)	
Age, year.	62 (23)	53 (19) ^**a,e**^	66 (25) ^**a**^	60 (24)	64 (23) ^**e**^	0.02
Male (*n*, %)	127 (78.4)	20 (74.1)	46 (71.9)	22 (84.6)	39 (86.7)	NS
BMI (kg/m^2^)	23.8 (6.1)	25.3 (10.0)	23.0 (5.1)	24.4 (5.5)	23.8 (5.9)	NS
Systolic blood pressure (mmHg)	106 (20)	106.0 (14.0)	108.0 (27.0)	100.0 (13.0)	104.0 (24.0)	NS
Diastolic blood pressure (mmHg)	60 (12)	58.0 (14.0)	60.0 (8.0)	60.0 (15.0)	60.0 (11.0)	NS
Heart rate (per min)	68 (17)	68.0 (11.0)	67.5 (16.0)	71.0 (15)	69.0 (15)	NS
LVEF (%)	33.9 ± 10.5	32.5 ± 8.4	36.6 ± 11.4	32.0 ± 8.7	31.9 ± 10.9	NS
NYHA class at polysomnography						NS
III, IV (*n*, %)	45 (27.8)	4 (14.8)	19 (29.7)	8 (30.8)	14 (31.1)	NS
Ischemic etiology (*n*, %)	53 (32.7)	6 (22.2)	19 (29.7)	7(26.9)	21 (46.7)	NS
History of HF (*n*, %)	89 (54.9)	14 (51.9)	38 (59.4)	16(61.5)	21 (46.7)	NS
AF (*n*, %)	57 (35.2)	7 (25.9)	26 (40.6)	8 (30.8)	16 (35.6)	NS
Diabetes (*n*, %)	49 (30.2)	4 (14.8)	23 (35.9)	5 (19.2)	17 (37.8)	NS
Laboratory data						
Hemoglobin (g/dL)	14.2 ± 2.5	14.7 ± 2.0	13.5 ± 2.8 ^**c**^	14.9 ± 2.0 ^**c**^	14.0 ± 2.1	<0.01
Albumin (g/dL)	3.6 (0.7)	4.0 (0.6) ^**a,e**^	3.3 (0.8) ^**a,c**^	3.8 (0.2) ^**b,c**^	3.4 (0.6) ^**b,e**^	<0.01
Lymphocyte count (per mm^3^)	1445.4 (938.2)	1925.0 (601.4) ^**a,e**^	1192.8 (890.5) ^**a,c**^	1782.8 (619.4) ^**b,c**^	1308.1 (1051.7) ^**b,e**^	<0.01
Total-cholesterol (mg/dL)	166 (51)	191 (35) ^**a,e**^	152 (55) ^**a,c**^	185 (55) ^**b,c**^	152 (36) ^**b,e**^	<0.01
Triglyceride (mg/dL)	93 (64)	142 (71) ^**a,e**^	77 (51) ^**a,c**^	102 (55) ^**b,c**^	78 (47) ^**b,e**^	<0.01
HDL-C (mg/dL)	42.3 ± 13.6	39.5 ± 14.6	44.5 ± 14.9	45.3 ± 11.7	39.0 ± 11.6	NS
LDL-C (mg/dL)	99 (41)	126 (40) ^**a,e**^	92 (46) ^**a,c**^	117 (53) ^**b,c**^	93 (26) ^**b,e**^	<0.01
Creatinine (mg/dL)	0.98 (0.55)	0.91 (0.30) ^**e**^	1.07 (0.70) ^**c**^	0.88 (0.33) ^**b,c**^	1.16 (0.61) ^**b,e**^	<0.01
eGFR (mL/min/1.73 m^2^)	58.1 ± 23.3	66.2 ± 18.5 ^**a,e**^	54.3 ± 18.7^**a,c**^	67.2 ± 27.2 ^**b,c**^	53.4 ± 23.1 ^**b,e**^	<0.01
CRP (mg/dL)	0.2 (0.5)	0.2 (0.4)	0.2 (0.5)	0.2 (0.3)	0.4 (0.7)	NS
BNP (mg/dL)	267 (332)	206 (257) ^**e**^	232 (293) ^**d**^	281 (312)	386 (644) ^**d,e**^	<0.01
CONUT, as continuous variable	2.0 (3.0)	1.0 (1.0) ^**a,e**^	4.0 (4.0) ^**a,c**^	1.0 (1.0) ^**b,c**^	3.0 (3.0) ^**b,e**^	<0.01
Medication						
Beta blockers (*n*, %)	154 (95.1)	26 (96.3)	60 (93.8)	25 (96.2)	43 (95.6)	NS
ACE-Is/ARBs (*n*, %)	135 (83.3)	23 (85.2)	52 (81.3)	24 (92.3)	36 (80.0)	NS
Aldosterone blockers (*n*, %)	110 (67.9)	20 (74.1)	40 (62.5)	17 (65.4)	33 (73.3)	NS
Loop diuretics (*n*, %)	132 (81.5)	21 (77.8)	46 (71.9)	23 (88.5)	42 (93.3)	0.02
Statin (*n*, %)	73 (45.1)	13 (48.1)	30 (46.9)	7 (26.9)	23 (51.1)	NS
Sleep study findings						
Total sleep time, min	355.9 ± 85.0	368.1 ± 79.8	346.9 ± 80.0	380.1 ± 74.9	347.2 ± 101.5	NS
% of slow wave sleep, % of TST	6.4 (9.6)	9.5 (5.9)	4.6 (9.4)	4.0 (10.6)	4.5 (9.4)	NS
% of REM sleep, % of TST	15.8 ± 7.2	17.3 ± 7.6	14.9 ± 7.5	16.0 ± 5.3	16.2 ± 7.5	NS
Arousal index, event/h of sleep	28.5 (23.1)	22.1 (15.3)	25.0 (30.0)	32.8 (21.5)	33.4 (18.1)	NS
AHI, events/h of sleep	32.5 (33.4)	18.75 (26.7) ^**e,f**^	21.90 (31.2) ^**c,d**^	44.00 (25.5) ^**c,f**^	42.10 (20.0) ^**e,d**^	<0.01
OAHI, events/h of sleep	11.3 (19.9)	8.60 (15.5)	13.50 (27.7)	10.22 (19.3)	12.35 (15.6)	NS
CAHI, events/h of sleep	12.2 (19.8)	12.35 (15.6) ^**e,f**^	7.38 (14.6) ^**c,d**^	19.23 (30.0) ^**c,f**^	24.10 (22.9) ^**e,d**^	<0.01
Mean SO_2_, %	95.2 ± 2.0	95.3 ± 2.0	95.6 ± 2.2	95.0 ± 2.2	95.2 ± 1.7	NS
PAP usage (*n,* %)	49 (30.2)	11 (40.7)	14 (21.9)	9 (34.6)	15 (33.3)	NS
CSR (*n,* %)	71 (43.8)	0 (0.0)	0 (0.0)	26 (100.0%)	45 (100.0%)	<0.01

Variables are expressed as mean ± standard deviation, median (interquartile range), or *n* (%). ACE-I, angiotensin-converting enzyme inhibitor; AF, atrial fibrillation; ARB, angiotensin II receptor blocker; AHI, apnea-hypopnea index; BMI, body mass index; BNP, B-type natriuretic peptide; CAHI, central apnea-hypopnea index; CSR, Cheyne-stokes respiration; CONUT, controlling nutritional status; eGFR, estimated glomerular filtration rate; HF, heart failure; LVEF, left ventricular ejection fraction; NYHA, New York Heart Association; OAHI, obstructive apnea-hypopnea index; PAP, positive airway pressure; REM, rapid eye movement; SO_2_, arterial oxyhemoglobin saturation; TST, total sleep time; NS: not significant. * Comparisons among four groups. **^a^**, significant difference between Non-CSR without malnutrition and Non-CSR with malnutrition; **^b^**, significant difference between Non-CSR with malnutrition and CSR with malnutrition; **^c^**, significant difference between Non-CSR with malnutrition and CSR without malnutrition; **^d^**, significant difference between Non-CSR with malnutrition and CSR with malnutrition; **^e^**, significant difference between Non-CSR without malnutrition and CSR with malnutrition; **^f^**, significant difference between Non-CSR without malnutrition and CSR without malnutrition; all *p* < 0.05.

**Table 2:** Univariate and Multivariate Cox regression analysis on all-cause mortality.

Characteristics	Univariate Analysis			Multivariate Analysis (Model 1)			Multivariate Analysis (Model 2)		
	HR	95% CI	*p*	HR	95% CI	*p*	HR	95% CI	*p*
Log-transformed age (1 increase)	4.45	0.77–25.58	NS	2.42	0.39–15.09	NS	2.56	0.41–16.15	NS
Log-transformed creatinine (1 increase)	2.45	1.49–4.04	<0.01	2.67	1.46–4.89	<0.01	2.73	1.47–5.06	<0.01
Log-transformed BNP (1 increase)	1.75	1.13–2.72	0.01	1.35	0.91–2.00	NS	1.34	0.91–1.99	NS
CSR—yes	2.58	1.15–5.79	0.02	3.92	1.64–9.39	<0.01			
CONUT (with malnutrition)	6.31	1.49–26.75	0.01	5.77	1.33–25.00	0.03			
Non-CSR/without malnutrition	Reference						Reference		
Non-CSR/with malnutrition	3.09	0.39–24.76	NS				2.10	0.25–17.10	NS
CSR/without malnutrition	0.88	0.06–14.03	NS				0.97	0.06–15.63	NS
CSR/with malnutrition	10.76	1.42–81.35	0.02				9.30	1.23–70.47	0.03

Model 1 includes age, creatinine, BNP, CSR and CONUT. Model 2 includes age, creatinine, BNP and the combined group of CSR and malnutrition. BNP, B-type natriuretic peptide; CSR, Cheyne-stokes respiration; CONUT, controlling nutritional status; NS, not significant.

## Data Availability

The data supporting this study’s findings are available from the corresponding author, upon reasonable request.
